# Raising sons or daughters for old age? Influence of children's gender on intergenerational family support in rural families

**DOI:** 10.3389/fpubh.2022.1063390

**Published:** 2023-01-04

**Authors:** Zhu Jiguang, Wang Yuncan, Song Yunxing

**Affiliations:** ^1^College of Economics, Henan University of Economics and Law, Zhengzhou, China; ^2^Academician Laboratory for Urban and Rural Spatial Data Mining, Henan University of Economics and Law, Zhengzhou, China; ^3^Department of City and Regional Planning, University of North Carolina at Chapel Hill, Chapel Hill, NC, United States

**Keywords:** raising sons for old age, raising daughters for old age, children's gender, size of family, intergenerational support

## Abstract

**Background:**

Under the background of miniaturization of family size and a growing number of young and middle-aged population outflow in rural China, the study of family pension mechanism in rural China from the perspective of changes in the pension functions of son and daughter will not only help to deepen the understanding of the change rules of China's family system, but also provide important reference for the future design of rural pension system.

**Data and method:**

The data come from the China Family Tracking Survey (CFPS), a nationwide social survey project runs by the Social Science Research Centre of Peking University. After excluding missing data, we obtained a valid sample of 11,207 sons and 2028 daughters in four data periods. We applied a fixed effects model for the analysis.

**Results:**

In rural areas, sons mainly provide economic support, while daughters mainly provide life care, thus forming a gender-based division of labor. With increasing off-farm job opportunities for daughters, they provide more economic support for their parents, but the time they spend on housework for their parents is reduced. As the number of children in a family has increased, daughters' role in supporting their parents has decreased. This research shows that although the traditional son-centered pension mode in China has not completely disintegrated, it has changed significantly. The findings reveal that changes in family size and improvements in women's status are important factors in changing family support patterns.

**Discussion:**

Different from the thought research about intergenerational relationship for a whole model, this article from the family internal different subjects role identity, shows the characteristics of the individual in the family, is conducive to theoretically explore the tension in the intergenerational relationship, individual and family which is helpful to understand the contemporary China's rural family generation ethics and intergenerational solidarity model. Families are classified more carefully according to the number, size and gender of children in the family, so as to fully show the heterogeneity and complexity of intergenerational relationships and old-age care models in rural families with different structural types. The discussion of the above issues has refined the description of rural family pension resources in China, which has certain reference significance for improving rural pension policies and actively dealing with the aging population.

## 1. Introduction

The concept of “raising sons for old age” exists in traditional rural China. With the aging of China's population and the economic and social changes in rural areas, the role of daughters in the family pension has gradually increased and has become a major trend in modern rural areas. The mode of “daughter support” not only breaks the division of labor in traditional family support, with sons as the core, but also reshapes the division of responsibilities in rural families. An increasing number of studies suggest that daughters are better than sons at providing economic support and life care (1–3). The new concept of daughter pension has been gradually accepted and recognized in rural societies; thus, daughter pension has become a new element of China's rural social pension pattern.

At present, academic research on rural old-age care in China mainly focuses on family old-age care, family structure, traditional gender concepts and support mechanisms.

Due to the lack of social security in traditional rural society, the family has assumed the main function of old-age care. In rural families in China and East Asia, children, especially sons, assume the responsibility of supporting their parents (4, 5). He et al. (6) asserted that the system of support for elderly people in rural China is based mainly on people providing feedback and caring for the old; indeed, in traditional farming society, single-endowment resources determine the dominant position of the family endowment (7). In traditional societies, production activities depend mainly on physical strength, and sons perform the dual function of producing labor and offspring. Therefore, bearing and rearing sons is considered a rational and less risky investment than raising daughters (8). Yin et al. (9) similarly found that in a society with relatively low productivity, because men are physically stronger than women and have more endurance and energy, people in China and East Asia are motivated to have boys.

In practice, the changes in the modern family structure have caused a situation in which daughters support their parents (10). As families have become smaller, the traditional role of sons as providers for their parents is no longer feasible to meet the true needs of rural social development, and daughters have also become providers to meet the needs of their elderly parents (11). Modern urban development has dismantled the traditional concept of filial piety (1), but at the same time, urbanization has improved the ability and willingness of daughters to support their parents and motivated them to do so. Horowitz (12) wrote that male migrant workers have contributed to the weakening of the concept of support, making daughters more important in providing for their parents and changing the nature of the family (13, 14). Sons who cannot directly assume the responsibility of supporting their parents provide economic support to their sisters, who in turn support their parents through the performance of certain household tasks (11).

The traditional concept of gender has also had a great impact on the ways in which children support their parents; it has been influenced by Confucianism, which holds that the purpose and meaning of having children is to provide “support”; i.e., sons are the most reliable resource for parents (15). Moreover, elderly people rely mainly on family members for their pension, and supporting parents in their old age is crucial (16). Ross and Alison (17) proposed the gender consciousness theory to explain that men play the role of material suppliers, while women play the role of family caregivers. When people raise children to support them in their old age and retirement, daughters do not play a role; instead, only sons play a role, as they are the most reliable resources for retirement (18). Preference for a certain gender (mainly for boys) has been a common phenomenon influencing fertility intentions in Chinese society (19). Most farmers have a strong fertility value orientation toward boys (20). Among people's reproductive objectives, the most important factors are the traditional concept of family legacy and the fact that people raise children so that these children can provide for them when they are old; these objectives constitute the root cause of people's universal preference for boys (21). In this paper, parents' preference for boys is based mainly on the traditional concept of family legacy and parents' practical need for support in their old age. People believe that having more children will make them happier, although it is mainly sons who will provide them with security in their old age; parents do not have great expectations of their daughters (22), and the responsibility of sons to support their parents leads parents to prefer their sons (21). This preference for boys over girls often leads to discrimination against daughters in parenting and intergenerational communication (23, 24).

Other scholars have found obvious differences between sons and daughters in terms of the support mode, time spent and rules that apply to them (3); specifically, daughters tend to care for parents' life needs and emotional support, and sons tend to provide economic assistance (25). Zeng et al. (25) found that due to gender-based psychological and physical differences, daughters often play a more practical role than sons in spiritual comfort and life care, which is more relevant to their parents' retirement needs. In addition, traditional principles dictate that the reciprocal relationship between generations emanates mainly from the Confucian principle of “reciprocating kindness”; that is, children support their parents in return for their parents' kindness in raising them Yan (26). When distinguishing the motivations of daughters and sons to support their parents, it has been found that the motivation of sons is based on “responsibility,” while that of daughters is based on “emotion” (25). Hence, daughters' support has become increasingly instrumental in current support practices in rural areas, and daughters have contributed immensely to meeting their parents' needs for life care and emotional support (2).

As rural areas have modernized, traditional concepts have continuously changed. The process of rural modernization has blurred the differences between genders in social production and narrowed the gender gap in intergenerational support (27). Daughters have assumed an increasingly prominent role in supporting their parents (10, 28, 29). Silverstein et al. (30) stated that daughters may be more filial than sons and more willing to take care of their elderly parents. The new concept of daughters' support for their parents has gradually been recognized by some villagers (31). Today, the idea of raising children to provide for old age and equating more children with more happiness has also changed; having children generally no longer refers to having sons (13, 32). The promotion of the role of daughters as providers for their parents has depended on the weakening of traditional gender systems and concepts (33) and the development of a modern consciousness (34).

In addition, spiritual support is directly related to elderly people's health, quality of life and happiness. As society has developed, the needs of older people in rural areas have changed from food and clothing to spiritual fulfillment. The needs of elderly people in rural areas are not limited to material needs, such as food, clothing and warmth, but also include spiritual comfort. Studies have found that daughters provide more emotional and functional support to their elderly parents than sons do (35, 36). The number of children in a family also affects the welfare of elderly individuals, as it is difficult for an only child to effectively meet the emotional needs of his or her parents (37).

Scholars in China and abroad have widely studied old age in rural areas and obtained relatively rich findings. However, research on rural pensions has fallen short. First, most current research has focused on family pensions as a whole. However, is there any significant difference in the level of support for parents' pensions provided by children within the same family based on gender? How does the gender of children affect the economic support and living care provided to parents in their old age? Second, research on family pensions has focused mainly on economic support, and the difference between genders in how much life care is provided remains unclear. Considering the improvement in women's economic and social status, what is their current status and role in supporting their parents? Can women promote a division of labor among children supporting their parents and improve the level of rural family pensions? In this paper, we use “gender role theory” and “resource endowment theory” to combine economic support with life care and, from the perspective of family support, explore whether and what kinds of differences exist in children's support for their parents as well as the change mechanism that causes gender-based differences in children supporting their parents.

## 2. Analytical framework and research hypothesis

**Figure 1:**
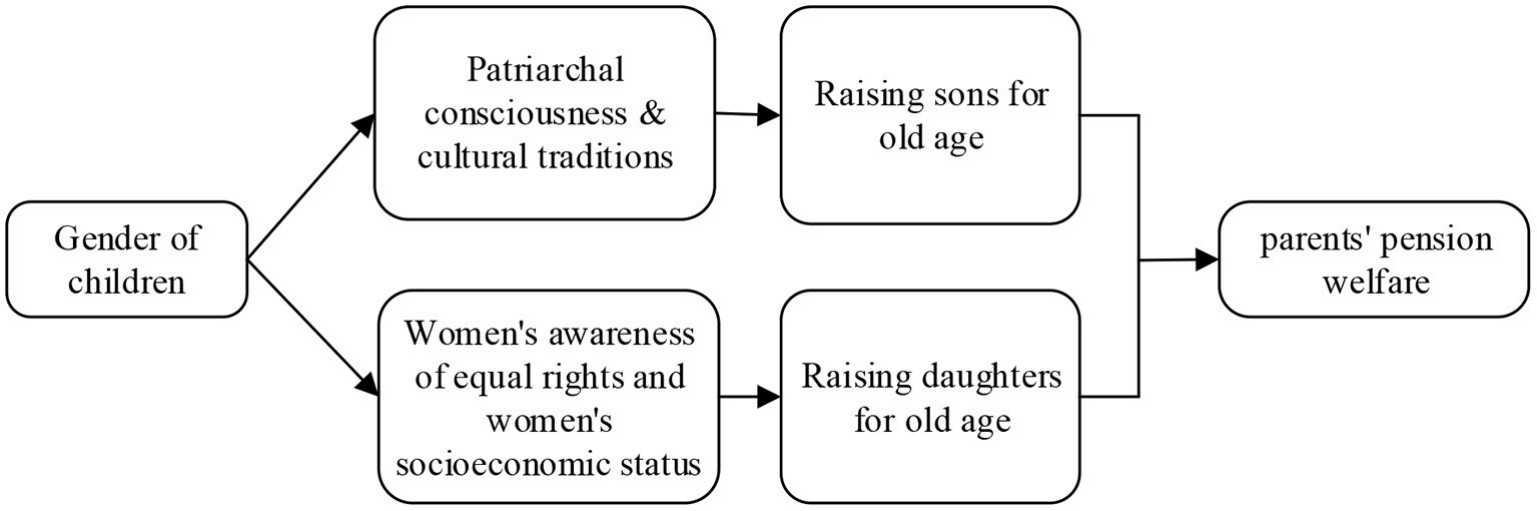
Theoretical research framework diagram.

Parents' pension benefits are realized in two ways (Figure 1). The first is that the son is the main provider of the parents. The son is considered the single resource for the parents' pension in traditional society, dictating the level of the parents' pension welfare. The second is that daughters provide important support to their parents. In traditional societies, sons and daughters act as a single resource for their parents' pension and jointly decide the level of parents' pension welfare through a specific family division of labor.

### 2.1. Gender-based differences in children's support of their parents

As the structure of the rural population has changed and rural modernization has accelerated, the gender lines in social production have gradually blurred, and the gender gap in children's intergenerational support has also narrowed (27). As a result, gender-based differences in social production have become increasingly unclear. Some studies have found that with the increasing role of daughters in family support, the gender gap in providing for parents has gradually also narrowed, although it remains significant (33). In rural areas, it is traditional to for couples have children as security for their old age, and this idea is still deeply rooted in people's hearts (22). Xu (11) found that daughters in rural families participate in their parents' retirement activities, but sons still play a greater role in their parents' pensions than daughters do. In addition, as the gap between children's education level and income has narrowed, men and women have become more equal. In rural areas, even though the level of support provided by daughters has improved, traditional ideas about having children for security in old age and “having more children for more happiness” have remained influential. Accordingly, we propose the following hypothesis.

Hypothesis 1: In rural families, there is still a traditional pension model based on sons being the main providers, and sons contribute more than daughters to their parents' pensions, but the gap between sons and daughters is narrowing.

### 2.2. Process by which children have changed the way they support their parents

In rural areas of China, all sectors of society have paid extensive attention to the fact that women are now supporting their parents. Two aspects of the narrowing of the gender gap in how children support their parents have mainly been considered: on the one hand, the role of men in supporting their parents has weakened, and on the other hand, the role of women in supporting their parents has increased. The second aspect is the most important, so this study focuses on socioeconomic status, education level, number of children in the family and other aspects to explore the impact of the gender gap on the ways in which children support their parents.

First, we consider the impact of children's socioeconomic status on parental support. Children's socioeconomic status directly affects the level of support they provide to their parents and is generally positively correlated with the economic support that they provide Zhang and Luo (38). Studies have found that in rural families, a certain gap remains between daughters and sons in terms of the level of economic support that they provide to their parents, but this gap is expected to gradually narrow as daughters' socioeconomic status improves (39). With adjustments in the industrial structure and the development of the tertiary industry, people no longer obtain income from wages for manual labor, and the sources of women's income, which has improved, have changed. These changes in income sources have led to daughters providing more support to their parents.

Second, the role of education as a relatively scarce resource allowing children to support their parents should not be underestimated. The more resources children have, the more likely they are to provide additional support (40, 41). Studies have found that the higher the children's education level, the greater their role in supporting their parents (26) and that offspring with higher education levels can provide more resources to their parents' pensions. Some scholars have conducted quantitative analyses using children's number of years of education to determine whether there are significant differences in how children support their parents based on education level. The results indicated that when children have 17 years of education, i.e., they have gone to college, there is no significant difference in the level of economic support provided by sons and daughters (11). Other studies have found that when daughters have studied for 12 years, i.e., they have finished high school, there is also no significant difference in the amount of care and support sons and daughters provide to their parents; moreover, studies have found that as education levels continue to increase, the role of daughters in caring for parents could exceed the role of sons (33).

Finally, the number of children in rural families not only affects the pension resources available to the elderly, but also is the basis for the division of pension responsibilities among children in such families (42, 43). The division of labor among children is the result of family members' subjective demands and emotional relationship negotiation under the restriction of the external environment. With the process of modernization and the decline of patriarchal system, daughters play a more and more prominent role in the family pension; thus, daughters are becoming increasingly involved in supporting their parents. However, the daughter's pension responsibility in a family with many children is more of an auxiliary responsibility. Parents and society have relatively low expectations for daughters regarding support of the elderly in families with many children, which may lead to the “mutual discharge of responsibilities” among children in rural families with many children, resulting in “free riding” behavior, thus reducing the level of parents' pension welfare.

In summary, in this paper, the following hypotheses are put forward.

Hypothesis 2: The higher a daughter's socioeconomic status is, the greater level of support she provides.
Hypothesis 3: The more educated a daughter is, the more support she will provide.
Hypothesis 4: The proportion of support provided by daughters decreases in families with more children.

## 3. Data sources and descriptive statistics

### 3.1. Data description

In this paper, we use data from the Chinese Household Tracking Survey (CFPS), a comprehensive nationwide social survey project organized by the Center for Social Sciences Survey at Peking University. The project adopts a sampling method, covering individual-level, family-level and social-level statistics, and the sample data cover 25 provinces, municipalities and autonomous regions in China (all except Hong Kong, Macao and Taiwan, Hainan, Qinghai, Tibet, Xinjiang, Inner Mongolia and Ningxia). In the survey, 2010 is the baseline, with surveys conducted every 2 years. To date, five periods of data have been published: 2010, 2012, 2014, 2016 and 2018. In this paper, we use four periods of panel data: 2010, 2012, 2016 and 2018. Data for 2014 are excluded because no survey was conducted that year on the parent-child relationship.

The CFPS data include information on financial support and household care provided by multiple children to elderly individuals, laying a foundation for in-depth study of variations in the level of support that children provide to their parents. As minor children are not economically able to support their parents, the sample in this research includes rural elderly people who are over 60 years of age and have children who are older than 18. In addition, because the data for each family include rural elderly individuals and their spouses, to avoid repeated calculations, we retain in the sample only one person older than 60 from each family. After eliminating data for which values are missing, we obtain 11,207 valid samples in the four data periods: 9,179 sons and 2,028 daughters.

In this paper, the core explained variables are “whether children provide their parents with economic support” and “whether children help their parents with housework and take care of their parents,” both of which are dichotomous. In this paper, the core explanatory variable is children's gender. In addition, the model controls for the children's age, marital status, years of education and type of occupation. The specific index system is shown in Table 1.

**Table 1:** Indicator system.

**Variable name**	**Variable symbol**	**Description of indicator assignment**
Economic support	*eco*	Whether children provide financial support to their parents: The value is 1 for yes and 0 for no.
Life care	*lif*	Do children help their parents with housework and care for their parents? The value is 1 for yes and 0 for no.
Children's gender	*gen*	For sons, the value is 1; for daughters, the value is 0.
Marriage	*mar*	When individuals are married, the value is 1; when they are not married, the value is 0.
Age	*age*	People's actual age
Education	*edu*	Years of education: illiteracy has a value of 0; primary school has a value of 6; junior high school has a value of 9; high school has a value of 12; junior college assignment has a value of 15; university education has a value of 16; a master's degree has a value of 18; and a PhD has a value of 22.
Number of children	*siz*	The actual number of children
Non-farm work	*nag*	Individual is employed in farm work: yes has a value of 1; no has a value of 0.
Agricultural production	*agr*	Individual is engaged in agricultural production: yes has a value of 1; no has a value of 0.
Household registration	*huj*	An urban hukou has a value of 1; a rural hukou has a value of 0.

### 3.2. Descriptive statistics

Based on our research sample, we perform a descriptive analysis of the main characteristics of daughters and sons in rural families. As shown in Table 2, overall, in rural families, the average number of years of sons' education is slightly higher than that of daughters, the proportion of sons engaged in non-farm work is 7.35% greater than that of daughters, and the unemployment rate among sons is 7.58% lower than that among daughters. The preference of rural families for having boys is partly corroborated by the fact that when a resource, such as education, is scarce, parents prioritize the needs of their sons. On the one hand, there is a certain positive correlation between the years of education and the type of occupation; that is, the more years of education, the higher the probability of engaging in non-farm work. On the other hand, our results show that in urban families, the preference for having boys has decreased.

**Table 2:** Descriptive statistics of children's characteristics.

**Variable**	**Categories/Indicators**	**Rural**			**Urban**		
		**Daughter**	**Son**	**Sum**	**Daughter**	**Son**	**Sum**
Age (in years)	Mean	38.27	41.02	40.63	38.93	41.44	40.79
	Standard deviation	9.97	8.66	8.90	9.26	8.91	9.07
Education (in years)	Mean	7.23	7.56	7.51	12.17	10.77	11.14
	Standard deviation	4.73	3.89	4.02	4.07	4.05	4.10
Married (%)	No	22.89	17.02	17.84	30.89	17.48	20.95
	Yes	77.11	82.98	82.16	69.11	82.52	79.05
	No work	26.82	19.24	20.29	34.65	34.62	34.63
Type of occupation (%)	Non-farm work	39.59	46.94	45.91	64.46	62.85	63.27
	Agricultural work	33.60	33.83	33.79	0.89	2.52	2.10

The comparison of urban and rural areas indicates that the average number of years of education of urban children is 3.63 years higher than that of rural children, indicating that there is still a certain gap in educational resources and educational levels between urban and rural areas. Although children in cities on average' go to school longer, the proportion of children in cities who do not work is greater than the proportion of rural children who are unemployed by 14.34%. On the one hand, this trend may be due to the pressures of the rural pension system; children leave school early to go work and earn money, thus leading to a lower mean for each education period. Moreover, under life pressures and restricted by the lower number of years spent studying, children in rural areas mostly engage in physical labor; thus, their unemployment rate is low. The parents of children in urban areas are highly educated, and most of them have pensions; therefore, they have a certain ability to provide for old age care and reduce the economic burden of their children. In addition, because children in urban areas are relatively highly educated, they are more resistant to engaging in manual work; instead, they seek work in the “knowledge economy.” These factors combine to cause the proportion of children who are unemployed to be higher in urban areas than in rural areas.

As shown in Table 3, in rural areas, sons provide significantly more economic support than daughters, but sons provide significantly less homecare than daughters. This finding explains why sons constitute the core of the rural family resource system; this system has not completely collapsed because sons still provide a slightly higher level of economic support. However, daughters care for the elderly and attend to the housework more than sons do, which confirms the “gender role theory”; that is, sons provide for the family materially, while daughters play the role of household carer in the family.

**Table 3:** Mean and T value test of sons and daughters providing financial support and household care to their parents.

**Variable**	**Sons**		**Daughters**			
	**Sample size**	**Mean**	**Sample size**	**Mean**	**Difference**	***T*** **test**
Economic support	9,179	0.282	2,028	0.267	0.015	2.041^**^
Life care	9,179	0.241	2,028	0.269	−0.028	2.812^***^

^**^p < 0.05, ^***^p < 0.01.

## 4. Basic regression analysis

### 4.1. Model setting

Our objective is to determine whether children's gender within the same family results in significant differences in the ways in which they support their parents. We use the fixed effects model for the analysis because this model can use information about multiple children in rural areas to compare the impact of their gender within the same family on the extent to which they contribute to their parents' support and welfare. When the dependent variable is dichotomous, the fixed effects model is as follows:


$$\begin{array}{l} log_{it}\left(P_{ij}\right)=log\frac{P_{ij}}{1-P_{ij}}=\sum_{k=0}^{K}\beta_{k}x_{kj}+\alpha_{i} \end{array}$$

where the subscript *i* denotes the parent and the subscript *j* denotes the child. *P*_*ij*_ is the probability that child *j* of parent *i* provides support to his or her parents. *x*_*kj*_ is the core explanatory variable for the child's gender, and α_*i*_ denotes the fixed coefficient of parent *i*, the heterogeneity of parent *i* itself.

Unlike classical logistic regression, the fixed effect model adds the dummy variable of the fixed coefficient of parent *i*, indicating whether, when both parents are *i*, there is any difference in children's support behavior. Therefore, through this model, we can more rigorously analyse trends within the same family. Although not for the parents after joining α_*i*_ level variables (such as age, marriage, and health), these variables are the same for children within the same family. Therefore, there is no need to control the level variables for parents again.

### 4.2. Basic regression results and analysis

When other variables remain unchanged, the economic support received by parents from their sons is significantly positive at the significance level of 10% (Table 4), which indicates that sons' economic support is significantly greater than daughters'. In contrast, the extent to which sons provide domestic care to their parents is significantly negative at the 1% level (Table 4), indicating that sons play a lesser role than daughters in supporting their parents.

**Table 4:** Effects of children's gender structure on intergenerational family support.

**Variable**	**Economic support**		**Life care**	
	**Coefficient**	**Standard error**	**Coefficient**	**Standard error**
*gen*	0.105^*^	0.057	−0.160^***^	0.058
*mar*	0.117^**^	0.057	0.074	0.059
*age*	−0.035^***^	0.003	−0.005^*^	0.003
*edu*	−0.009^*^	0.005	−0.001	0.006
*nag*	−0.046	0.059	0.097	0.064
*agr*	−0.110	0.068	0.438^***^	0.070
*Constant term*	−0.144	0.145	−0.609^***^	0.145
*year*	Yes		Yes	
Sample size	11,207		11,207	
Likelihood ratio chi-square	351.697^***^		464.410^***^	
*R^2^*	0.027		0.037	

^*^p < 0.1, ^**^p < 0.05, ^***^p < 0.01.

In rural families with both sons and daughters, the sons mainly provide economic support to their parents, while daughters mainly provide household care. The historical principles of traditional rural society in China hold that parents should raise children for security in old age and that “having more children brings more happiness.” However, the son-centered support mechanism has changed when it comes to household care, as daughters perform more of this care, and daughters have also begun to participate in activities that support their parents; hence hypothesis 1 is verified. In rural areas, elderly men are generally supported by their sons and daughters-in-law. However, elderly men and daughters-in-law lack a common emotional foundation, and the subtle “old woman daughter-in-law relation” means that daughters-in-law can meet support requirements only to a certain extent. Elderly men's sons, because they have left the area to seek work, often provide economic support and ignore spiritual care. Therefore, elderly people prefer to receive spiritual care from their daughters. Although daughters are less likely to provide financial support to their parents after marriage, their intimate, sensitive feelings and shared experience of having children enable them to better understand their parents' hardships; thus daughters have an advantage over sons and daughters-in-law in terms of providing spiritual support. When parents need care, daughters play an indispensable role of companionship and spiritual comfort. In other words, people's preference for having boys to secure their old age in rural society is not one-sided but is based on both genders' preferences.

## 5. Mechanism of the effect of children's gender on parental support

According to the above studies, in China's vast rural society, traditional support mechanisms have endured: sons provide “support,” but daughters have begun to be involved in providing support to their parents. Hence, we further analyse the differences between urban and rural areas, changes in the socioeconomic status of women and number of children to understand how children support their parents.

### 5.1. Urban–rural differences

To study differences between urban and rural areas in the ways in which children support their parents, we consider the interaction between urban and rural household registration status and children's gender on the basis of our original model, and the results are shown in Table 5.

**Table 5:** Estimated results of urban–rural differences based on children's gender and level of parental support.

**Variable**	**Economic support**		**Life care**	
	**Coefficient**	**Standard error**	**Coefficient**	**Standard error**
*gen*	0.123	0.077	−0.202^***^	0.077
*gen × huj*	−0.106	0.115	0.072	0.116
*huj*	−0.155	0.106	−0.149	0.108
*mar*	0.111^*^	0.057	0.068	0.060
*age*	−0.033^***^	0.003	−0.004	0.003
*edu*	−0.001	0.006	0.002	0.006
*nag*	−0.095	0.060	0.080	0.065
*agr*	−0.214^***^	0.072	0.398^***^	0.074
*Constant term*	−0.168	0.150	−0.577^***^	0.150
*year*	Yes		Yes	
Sample size	11,207		11,207	
Likelihood ratio chi-square	373.166^***^		467.552^***^	
*R^2^*	0.028		0.037	

^*^p < 0.1, ^***^p < 0.01.

The interaction between children's gender and household registration is not significant in terms of how much economic support children provide for their parents' pension or household care, which indicates that there is no significant difference between sons and daughters in supporting their parents in the urbanized countryside. As populations in urban and rural areas have become increasingly more mobile, caring for older people is becoming integrated across urban and rural areas. Hence, children support their parents equally in urban and rural areas. However, some studies have shown significant differences between urban and rural areas in the extent to which children support their parents (44). First, this significant difference is due to the obvious income gap between urban and rural areas, which makes urban children better able than rural children to provide economic support to their parents. Second, different conceptualisations of old-age care lead to certain differences between urban and rural areas in how children support their parents. People in rural areas are relatively conservative and have always adhered to the concept that they should raise children for more security in their old age, resulting in sons being the key to their parents' support in those areas.

### 5.2. Women's status

In general, there is a positive correlation between women's socioeconomic status, the number of years they spend in school and their income level. That is, the more educated women are, the higher their status is in their family and society. Moreover, improvements in women's socioeconomic status will raise their income level and help them provide more economic support to their parents. To study whether the socioeconomic status of daughters has an impact on the ways in which they support their parents, we successively include two cross-terms in the model: gender and non-farm work and gender and years of education.

As shown in Table 6, the effect of the coefficient of the cross-term between children's gender and off-farm work on economic support is significantly negative, indicating that participation in off-farm work significantly increases daughters' economic support to their parents. On the one hand, the reason for this finding may be that off-farm work enhances the income level of daughters, thus laying the economic foundation for them to support their parents. On the other hand, daughters' traditional views change consciously or unconsciously after they engage in off-farm work, laying the sociocultural foundation for them to support their parents. Combined, these two factors make daughters who take part in off-farm work more likely to provide economic support to their parents. Therefore, hypothesis 2 is verified. However, the cross-coefficient that pertains to housework is 0.149, and there are no statistically significant results. When both sons and daughters work in non-farm jobs, their ability to take care of their parents' housework will not be improved. Because time is a scarce resource, extended working hours reduce the ability of sons and daughters to provide support to their parents. In summary, a daughter's family and social status improves when she takes part in non-farm work, and her ability to provide economic support to her parents is enhanced.

**Table 6:** Estimated results of the impact of children's socioeconomic status on parental pension.

**Variable**	**Economic support**		**Life care**	
	**Coefficient**	**Standard error**	**Coefficient**	**Standard error**
*gen*	0.240^*^	0.127	−0.287^**^	0.122
*gen × nag*	−0.314^**^	0.128	−0.149	0.130
*gen × edu*	0.001	0.013	0.021	0.013
*huj*	−0.247^***^	0.053	−0.092	0.056
*mar*	0.111^*^	0.057	0.067	0.060
*age*	−0.033^***^	0.003	−0.004^*^	0.003
*edu*	−0.002	0.012	−0.015	0.012
*nag*	0.157	0.119	0.199	0.121
*agr*	−0.235^***^	0.072	0.388^***^	0.075
*Constant term*	−0.250	0.172	−0.499^***^	0.168
*year*	Yes		Yes	
Sample size	11,207		11,207	
Likelihood ratio chi-square	379.660^***^		469.981^***^	
*R^2^*	0.029		0.038	

^*^p < 0.1, ^**^p < 0.05, ^***^p < 0.01.

Second, the coefficients of the cross-term of children's gender and years of education for economic support and household care are both positive but non-significant. Generally, children's education level is positively correlated with the extent to which they support their parents; that is, children with higher education levels are more likely to provide support. Previous studies have shown that when children have 17 years of education (college), there is no significant difference in the degree of economic support between sons and daughters, and when children have 8 years of education (junior high school), the difference in the support provided by sons and daughters in the form of household care disappears (11). One reason for our results may be that in our sample, only 14% of children have a college education or above, while 86% have an education level that lower than high school. When comparing results across education levels, we find that basic education has little effect on income; thus, the education level of children has little influence on the parents' retirement. This conclusion does not support hypothesis 3.

### 5.3. Number of children

The number of children has a certain impact on their support of their parents. Therefore, the cross-term between the gender of children and the number of children in a family is included in the analytical model. The result of the cross-term represents the impact of the number of children in a family on the ways in which these children support their parents.

As shown in Table 7, the coefficients of the interaction terms between children's gender and the number of children in a family are both positive and significant at the 1% level in terms of economic support and household care, indicating that in families with many children, sons play a relatively greater role in providing economic support and household care to their parents. The reason may be that in rural areas, people generally believe that it is “legitimate” and in line with tradition for sons to support their parents, so there is a greater expectation that sons will support their parents. Since people in rural areas believe that raising children provides security for their old age, it is not surprising that within the same family, sons provide more financial support and household care to their parents. After they marry, daughters are considered to be “spilt water.” Parents expect little from daughters in terms of support because after marriage, daughters, along with their husbands, mostly serve their in-laws. If married women provide financial support to their parents and take care of housework for them, it is bound to cause dissatisfaction among their in-laws and conflict that is not conducive to family harmony. In addition, in rural families in which there are many children, children compete to show how much they support their parents, playing an exemplary role to improve the overall level of support they provide. However, when sons provide more economic and housework care, some daughters may be “free riders” and reduce the financial support and household care that they provide to their parents. The results show that in families with many children, sons provide more financial support and housework than daughters. Therefore, hypothesis 4 is supported.

**Table 7:** Estimated results of the influence of family size on children's contribution to their parents' care.

**Variable**	**Economic support**		**Life care**	
	**Coefficient**	**Standard error**	**Coefficient**	**Standard error**
*gen*	−0.170^***^	0.063	−0.329^***^	0.064
*gen × siz*	0.178^***^	0.018	0.115^***^	0.019
*huj*	−0.210^***^	0.053	−0.074	0.056
*mar*	0.141^**^	0.057	0.090	0.060
*age*	−0.034^***^	0.003	−0.005^*^	0.003
*edu*	0.004	0.006	0.005	0.006
*nag*	−0.068	0.060	0.093	0.065
*agr*	−0.203^***^	0.072	0.405^***^	0.074
*Constant term*	−0.133	0.146	−0.607^***^	0.145
*year*	Yes		Yes	
Sample size	11,207		11,207	
Likelihood ratio chi-square	467.446^***^		504.464^***^	
*R^2^*	0.036		0.040	

^*^p < 0.1, ^**^p < 0.05, ^***^p < 0.01.

## 6. Tests for robustness and endogeneity

### 6.1. Robustness tests

To test the robustness of our results, we used the propensity score matching (PSM) method to retest the above model. The PSM method can alleviate the problem of biased estimation caused by the incorrect setting of functional forms by reducing the dependence on functional forms. The scores shown in Table 8 tend to match the balance to the test results. The absolute value of the standard deviation of all matching variables is <10%. The characteristics of the experimental group and control group are similar and match after matching the t value, and there are basically no significant variables. After matching the experimental group and control group, there is no significant difference in the balance after the data match. A total of 8,421 samples are obtained by the PSM method.

**Table 8:** PSM balance test.

	**Unmatched**	**Mean**			**% Reduction**	**T test**	
**Variable**	**Matched**	**Treated**	**Control**	**% Bias**	**|bias|**	**t**	**p**>**|t|**
*huj*	*U*	0.31528	0.49803	−37.9		−15.8	0.000
	*M*	0.30698	0.30668	0.1	99.8	0.04	0.970
*mar*	*U*	0.8283	0.73126	23.6		10.14	0.000
	*M*	0.89399	0.89108	0.7	97	0.54	0.590
*age*	*U*	41.149	38.602	27.7		11.66	0.000
	*M*	40.151	40.198	−0.5	98.2	−0.36	0.718
*edu*	*U*	8.57	9.6933	−24.1		−10.46	0.000
	*M*	8.7862	8.8172	−0.7	97.2	−0.44	0.659
*nag*	*U*	0.51956	0.51972	0		−0.01	0.989
	*M*	0.54718	0.54894	−0.4	−948.8	−0.2	0.838
*agr*	*U*	0.23957	0.17308	16.5		6.48	0.000
	*M*	0.2514	0.2511	0.1	99.5	0.04	0.968

The coefficient signs and significance of the core explanatory variables in each model in Table 9 are basically consistent with the basic regression results, indicating that the estimation results of this study have a certain robustness. Among them, the coefficients of the interaction terms between children's gender and urban and rural household registration based on economic support and household care are −0.135 and 0.031, respectively, which are not significant (column 2 and column 3 of Table 8). This result indicates that there is no significant difference between the level of support sons and daughters provide to their parents in the urbanized countryside, which is consistent with the results of the basic regression. To verify whether socioeconomic status affects the ways in which children support their parents, two interaction terms are added successively: children's gender and off-farm work and gender and children's years of education. The results show (column 4 and column 5 of Table 8) that the interaction term between sons and off-farm work is significantly negative at the 5% level. This result indicates that daughters' participation in off-farm work significantly increases the amount of economic support they provide to their parents, but the interaction term between gender and off-farm work is not significant in terms of household care, indicating that, once they participate in off-farm work, neither sons nor daughters provide more domestic care to their parents. This result is consistent with the results of the basic regression. The cross-terms of sons and years of education are not significant in terms of economic support and household care, which further indicates that the level of children's education has little influence on the support provided to their parents. To study the robustness of family size on the ways in which children support their parents, we add the interaction term between children's gender and family size and use the propensity matching score PSM method to conduct a fixed-effect regression. The results (column 6 and column 7 in Table 8) show that for families with many children, the coefficients of sons on economic support and household care are 0.196 and 0.103, respectively, and both are significant at the 1% level, indicating that the larger the family, the more financial support and household care son provide compared to daughters, which is consistent with the results of the basic model.

**Table 9:** Robustness estimation results for the effect of children's gender role on the ways in which they support their parents.

**Variable**	**Differences between**		**Socioeconomic status**		**Number of children**	
	**urban and rural areas**					
	**Coefficient**	**Standard error**	**Coefficient**	**Standard error**	**Coefficient**	**Standard error**
*gen*	0.105 (0.081)	−0.180^**^ (0.081)	0.250^*^ (0.139)	−0.288^**^ (0.135)	−0.221^***^ (−3.207)	−0.306^***^ (−4.368)
*gen × huj*	−0.135 (0.123)	0.031 (0.126)				
*gen × nag*			−0.285^**^ (0.140)	−0.085 (0.145)		
*gen × edu*			−0.004 (0.015)	0.018 (0.015)		
*gen × siz*					0.196^***^ (9.040)	0.103^***^ (4.566)
*huj*	−0.117 (0.113)	−0.115 (0.116)	−0.227^***^ (0.063)	−0.092 (0.067)	−0.184^***^ (-2.887)	−0.074 (−1.095)
*mar*	0.097 (0.076)	0.056 (0.080)	0.096 (0.076)	0.057 (0.080)	0.128* (1.687)	0.073 (0.912)
*age*	−0.032^***^ (0.004)	−0.003 (0.004)	−0.031^***^ (0.004)	−0.003 (0.004)	−0.033^***^ (−9.030)	−0.004 (−1.061)
*edu*	−0.001 (0.007)	−0.003 (0.008)	0.001 (0.013)	−0.016 (0.013)	0.003 (0.343)	−0.002 (−0.273)
*nag*	−0.105 (0.073)	0.182^**^ (0.080)	0.110 (0.129)	0.245^*^ (0.133)	−0.070 (−0.959)	0.198^**^ (2.480)
*agr*	−0.199^**^ (0.086)	0.494^***^ (0.090)	−0.226^***^ (0.086)	0.490^***^ (0.091)	−0.192^**^ (−2.234)	0.498^***^ (5.533)
*Constant term*	−0.229 (0.188)	−0.649^***^ (0.190)	−0.327 (0.206)	−0.567^***^ (0.205)	−0.182 (−0.982)	−0.656^***^ (−3.511)
*year*	Yes	Yes	Yes	Yes	Yes	Yes
Sample size	8,421	8,421	8,421	8,421	8,421	8,421
Likelihood ratio chi-square	266.691	390.330	271.716	391.737	346.618	410.872
*R^2^*	0.027	0.041	0.027	0.041	0.035	0.043

Standard error in parentheses; ^*^p < 0.1, ^**^p < 0.05, ^***^p < 0.01.

### 6.2. Endogeneity test

The endogenous problem between the core dependent variable and the independent variable must be avoided. According to the research methods of other scholars (45), the variable lags behind one period and is related to the current period. However, because the unobservable variable is determined in advance, the lag period of this variable is not related to the current disturbance term; thus, the subsequent period of the variable can effectively become a tool variable of the current period. In this study, the independent variable that lags one period is a tool variable. Table 10 shows the estimated results using the tool variables. First, tool variables were used to check whether there were endogenous variables present, and the effectiveness of the tool variables was evaluated. The results show that the endogenous test statistics reject the original hypothesis at the level of 1%, indicating that the endogeneity of the independent variable is significant; thus, the regression of the results using tool variables is more accurate. The test results of weak instrumental variables are all >10, indicating the absence of weak instrumental variable concerns. The above tests show that the tool variables selected in this paper are appropriate. The study found that sons have a positive but not significant impact on parents' economic support; son's life care for parents is significantly negative at the level of 5%, indicating that the daughter plays a significant role in parental life care.

**Table 10:** Test results of instrumental variables of children's parental support behavior.

**Variable**	**Economic support**	**Life care**
L_gen	0.002 (0.031)	−0.081^**^ (0.030)
Control variable	Yes	Yes
Weak identification test	1,812.762	1,812.762
Underidentification test	1,521.650	1,521.650

Standard error in parentheses, ^**^p < 0.05.

## 7. Discussion

Our research not only contributes to an in-depth understanding of the changes taking place in China's rural family pension strategies but also has implications for policy in today's aging China. First, the tradition in rural areas of raising children to gain security in old age has led parents to prefer having boys. However, studies have shown that daughters are playing an increasingly important role in supporting their parents, especially because they perform housework. Will this change lessen the preference for boys in rural families? How will this change affect the gender imbalance in rural areas? These questions are worth further academic attention.

Second, in this research, we select rural families with many children, thus avoiding the influence of individual parental differences on children's support behavior, which is consistent with the reality of most families with many children in rural China. Studies have shown that the larger the family, the more financial support sons provide; that is, when there are fewer children, daughters provide more support to their parents. This result is valid mainly when resources are scarce; hence, daughters make a passive choice, leading to more material and spiritual support for elderly individuals. However, as the birth rate continues to decline and the rural population continues to age, causing an increasing number of rural families to have only one child, the burden of family pension will further increase. The adjustment of the number of children in rural families alone is not a long-term solution to the problem of rural family old-age care. New models of old-age care, such as rural home care and community care, can not only meet the needs of elderly people in rural areas but also reduce children's burden of caring for elderly parents.

Finally, we consider the role of children's gender in the level of financial support they provide to their parents based on two factors, but due to limitations in the data, this paper has the following deficiencies. First, only in 2010 did the data show how far children live from their parents, and distance greatly affects children's level of support to their parents. Generally, children living close to their parents provide more support. Moreover, we do not consider daughters-in-law and their husbands in the family support system. In rural areas, married daughters traditionally join their husbands' families and, together with their husbands, are required to serve their in-laws, and sons-in-law, to a certain extent, participate in supporting their parents. However, because the data do not investigate the support provided by daughters-in-law and sons-in-law, it is difficult to distinguish the share of support provided by daughters and sons-in-law and sons and daughters-in-law. Therefore, the family support system needs to be further explored.

## 8. Conclusion

Based on the fourth set of panel data of the Chinese Family Tracking Survey (CFPS), we investigate the differences in the ways in which children support their parents and the mechanisms that influence this support in rural families from the perspectives of economic support and life care. We find that in rural areas, sons mainly provide economic support, while daughters mainly provide life care, thus forming a gender division of labor in rural areas. There is no significant difference between sons and daughters in rural areas and urban areas. Children's education level is positively related to the level of support they provide to their parents, but our regression results are not significant. With the increase in off-farm job opportunities, daughters provide more financial support to their parents but reduce the time they spend on housework for them. With the increase in the number of children in a family, daughters may free-ride, allowing others to support their parents, and their share of support decreases, while sons provide relatively more in terms of both economic support and household care. Our research shows that although the traditional son-centered support system in China has not completely disintegrated, it has changed significantly. In general, changes in the size of the population and improvements in women's status are important factors in explaining the changes taking place in the rural family support model.

This work provides three potential contributions. First, different from thought research regarding intergenerational relationships in a whole model, this article focuses on the role identity of internal subjects in a family and shows the characteristics of the individual in the family, which is conducive to theoretically explore tension in intergenerational relationships. Studying individuals and families helps explain contemporary China's model of rural family generation ethics and intergenerational solidarity (46). Most scholars believe that the function of rural family pension has been weakened; however, this study finds that the family pension model with children supporting the elderly as the core still occupies a very important position in rural areas. Different from previous studies, this paper finds that there is exclusion and substitution in economic support and life care provided by children in rural elderly populations. The probability of the rural elderly receiving economic support and life care from their children is relatively low. This finding is in line with the reality that the spatial distance between children and parents increases abruptly due to the transfer of the young and middle-aged rural labor force and the decreased opportunity for children to live with the elderly due to the transformation of the rural family core (47).

Second, families are classified more carefully here according to the number of children, family size and gender of children to fully show the heterogeneity and complexity of intergenerational relationships and old-age care models in rural families with different structural types. In the multi-child family supporting the elderly shown in this paper, daughters may demonstrate free-riding behaviors in terms of parental support, and their input in parental support is reduced, while sons play a relatively large role in both economic support and household care. Women provide emotional support and do not fully consider the economic ideal of the initiative to pay, reflecting the unique role of women in the family pension. In spite of reduced conventions in rural households, China's rural system is still not similar to the western individual framework, and families still follow traditional Chinese family roles. Sons remain in a dual system of ethics and moral emotion regarding specific financial responsibilities, representing intergenerational ethics and solidarity that differ substantially from western ideals. The change in family social status leads to different pension modes. With the improvement of women's social and economic status and the rationalization trend of “daughter pension” in public opinion and ethics, daughters gradually increase parental economic support and life care, and this support is considered altruistic with the concept of filial piety and gratitude. Sons are more likely to follow the traditional norms in rural areas. Supporting their parents is a reflection of following the traditional theory of filial piety and fulfilling their responsibility to support (48, 49).

Third, the discussion of the above issues has refined the description of rural family pension resources in China, which has certain reference significance for improving rural pension policies and actively dealing with the aging population. With the change in rural fertility concepts, the trend of the family core is enhanced. As for how to increase rural elderly care, the authors believe that the government, the market and the family multi-dimensional main body should coordinate and assistant in a complementary manner (46). Based on the trend of increasing intergenerational support provided by rural daughters to their parents, social gender preferences should be changed from a preference for sons to a social consensus that “adopted daughters can also protect their old age.” Finally, the government needs to increase the financial expenditure and resource input for rural elderly care through policies that provide public services and prioritize the groups with weak rural elderly care resources.

## Data availability statement

The raw data supporting the conclusions of this article will be made available by the authors, without undue reservation.

## Author contributions

SY: conceptualization. WY: methodology, data curation, and writing—original draft preparation. ZJ: formal analysis, writing—review and editing, and supervision. All authors have read and agreed to the published version of the manuscript.
